# The Development of Audio-Visual Integration for Temporal Judgements

**DOI:** 10.1371/journal.pcbi.1004865

**Published:** 2016-04-14

**Authors:** Wendy J. Adams

**Affiliations:** Psychology, University of Southampton, Southampton, Hampshire, United Kingdom; University of Birmingham, UNITED KINGDOM

## Abstract

Adults combine information from different sensory modalities to estimate object properties such as size or location. This process is optimal in that (i) sensory information is weighted according to relative reliability: more reliable estimates have more influence on the combined estimate and (ii) the combined estimate is more reliable than the component uni-modal estimates. Previous studies suggest that optimal sensory integration does not emerge until around 10 years of age. Younger children rely on a single modality or combine information using inappropriate sensory weights. Children aged 4–11 and adults completed a simple audio-visual task in which they reported either the number of beeps or the number of flashes in uni-modal and bi-modal conditions. In bi-modal trials, beeps and flashes differed in number by 0, 1 or 2. Mutual interactions between the sensory signals were evident at all ages: the reported number of flashes was influenced by the number of simultaneously presented beeps and vice versa. Furthermore, for all ages, the relative strength of these interactions was predicted by the relative reliabilities of the two modalities, in other words, all observers weighted the signals appropriately. The degree of cross-modal interaction decreased with age: the youngest observers could not ignore the task-irrelevant modality—they fully combined vision and audition such that they perceived equal numbers of flashes and beeps for bi-modal stimuli. Older observers showed much smaller effects of the task-irrelevant modality. Do these interactions reflect optimal integration? Full or partial cross-modal integration predicts improved reliability in bi-modal conditions. In contrast, switching between modalities reduces reliability. Model comparison suggests that older observers employed partial integration, whereas younger observers (up to around 8 years) did not integrate, but followed a sub-optimal switching strategy, responding according to either visual or auditory information on each trial.

## Author Summary

To complete everyday activities, such as judging where or when something occurred, we combine information from multiple senses such as vision and audition. In adults, this merging of information is optimal: more reliable sensory estimates have more influence (higher weight) in the combined, multisensory estimate. Multisensory integration can result in illusions: if a single visual flash (e.g. a bright disk appearing briefly on a screen) occurs at the same time as two beeps, we sometimes perceive two flashes. This is because auditory information is generally more reliable than vision for judging *when* things happen; it dominates our audio-visual percept for temporal tasks. Previous work suggests that children don’t combine information from different senses in this adult-like way until around 10 years. To investigate this further, we asked children and adults to report the number of visual flashes or auditory beeps when these were presented simultaneously. Surprisingly, all children used appropriate sensory weights: audition—the more reliable signal—tended to dominate perception, with less weight given to vision. However, children didn’t show the adult-like reduction in uncertainty until around 8–10 years. Before that age, they switched between using only auditory or only visual information on each trial.

## Introduction

Imagine you are at an academic conference. A heated debate turns nasty and one scientist is repeatedly hit before falling to the floor. You are later asked how many punches were thrown. You confidently answer ‘3’; you were able to combine information from audition and vision, having both seen and heard the incident. We often receive information about the same object or event from multiple sensory modalities that we can integrate to improve the precision of our perceptual estimates. As adults, we integrate multisensory information for a variety of spatial and temporal tasks, such as judging the size, location, number or duration of objects or events [1–5]. A key benefit of this integration is that uncertainty, or variance (random noise) in the combined, multisensory estimate is reduced, relative to either of the component uni-sensory estimates, see e.g. [6].

Under standard models of integration, sensory estimates are combined via weighted averaging, according to the estimates’ relative reliabilities, see e.g. [1, 2, 6]. For example, consider the case in which an aspect of the environment is estimated from vision and audition. The visual and auditory estimates, ${\hat{S}}_{V}$ and ${\hat{S}}_{A}$ are not perfectly precise, but contain noise with variance $\sigma_{V}^{2}$ and $\sigma_{A}^{2}$. It is commonly assumed that these noise distributions are Gaussian and independent. Under these assumptions, and given that the prior probability distribution over the estimated variable is uniform, then the optimal audio-visual estimate (i.e. that with the lowest possible variance), for a continuous variable is given by:

$${\hat{S}}_{VA}=w_{V}{\hat{S}}_{V}+w_{A}{\hat{S}}_{A}$$

with the visual and auditory weights, *w*_*V*_ and *w*_*A*_ defined as:

$$w_{V}=\frac{1/\sigma_{V}^{2}}{1/\sigma_{V}^{2}+1/\sigma_{A}^{2}}\text{ and }w_{A}=\frac{1/\sigma_{A}^{2}}{1/\sigma_{V}^{2}+1/\sigma_{A}^{2}}.$$

As can be seen from the equations above, sensory weights give the relative influence of each uni-modal sensory estimate in determining responses to bi-modal stimuli. These weights can be estimated from behavioural data corresponding to bi-modal and uni-modal stimulus conditions. For example, in a size estimation task such as [1], subjects might be required to estimate an object’s size from vision alone, from haptics (touch) alone, or from both vision and hatpics. If the visual size is 9cm and the haptic size is 12cm, then given unbiased uni-modal estimates, a mean bi-modal response of 10cm would correspond to visual and haptic weights of 2/3 and 1/3, respectively, i.e. vision has double the influence of haptics. These observed weights would be optimal if the uni-modal visual estimates were twice as reliable as the uni-modal haptic estimates, i.e. $\sigma_{V}^{2}/\sigma_{H}^{2}=0.5$.

Observing optimal sensory weights is consistent with optimal integration, i.e. the integration behaviour that minimises variance in the multimodal estimates. However, optimal sensory weights might be observed in the absence of integration: as an alternative to integration, an observer may select one of the uni-modal estimates on each trial, rather than computing a weighted average [7, 8]. In the example above, the observer may select the visual estimate on 2/3 of trials, and the haptic estimate on 1/3 of trials. This ‘switching’ behaviour would produce the same mean response in bi-modal conditions as optimal integration, but with higher variance. Standard models predict that variance will be reduced in bi-modal, relative to uni-modal conditions under optimal integration, see, e.g. [1, 2, 6, 9]. For example in the visual-haptic size example, under optimal integration the predicted variance of the visual-haptic estimates, $\sigma_{VH}^{2}$, is given by $\sigma_{VH}^{2}=\frac{\sigma_{V}^{2}\sigma_{H}^{2}}{\sigma_{V}^{2}+\sigma_{H}^{2}}$. In contrast, switching behaviour will result in variance that is at least as large as the more reliable cue. For this reason, studies of multimodal integration generally determine (i) whether the sensory weights are optimal, given uni-sensory variability, and (ii) whether variability in the bi-modal estimates is reduced, relative to uni-modal estimates.

Recently, a number of studies have asked whether children show optimal integration of sensory cues, as indexed by (i) appropriate cue weighting and (ii) a reduction in variance, relative to single cue conditions. Gori and Burr [10] reported that optimal integration of multisensory information doesn’t appear until surprisingly late—at the age of around 10 years. In two visual-haptic tasks, younger children who were asked to judge object size or orientation relied on only one modality, and not necessarily the most reliable one. Other work has confirmed that children as old as 8 years fail to optimally integrate visual cues with movement-based information (proprioceptive and vestibular) for navigation [7], and another study suggests that optimal integration of auditory and haptic information does not occur until after age 11 [11]. Interestingly, this developmentally late integration is not limited to situations in which information must be combined from different sensory modalities: Nardini and colleagues reported similarly late integration for cues within a modality—optimal integration of two visual depth cues did not emerge until around age 12 [12].

The current study focuses on the developmental trajectory of audio-visual integration, using a straightforward counting task. The age at which optimal integration emerges for vision and audition is not yet clear. One previous audio-visual study with children aged 5–14 years and adults failed to find optimal integration at any age [13]. We employed a simple audio-visual task in which, on each trial, observers were presented with a number of beeps and / or flashes [14]. In separate blocks, they either reported the number of flashes, or the number of beeps. The task had the benefit of reduced memory and decisional demands, relative to previous studies that have used two-alternative forced choice designs. By comparing observers’ responses to different integration models we ask:

1. Do children show optimal integration of auditory and visual information? If so, from what age?
2. Is integration mandatory? In our task, observers are asked to report only one modality or the other, i.e. either beeps, or flashes, rather than the number of audio-visual events. We ask whether children do ignore the irrelevant (non-reported) modality, and we determine whether the strength of cross-modal interactions changes as function of age.

## Results

**Fig 1:**
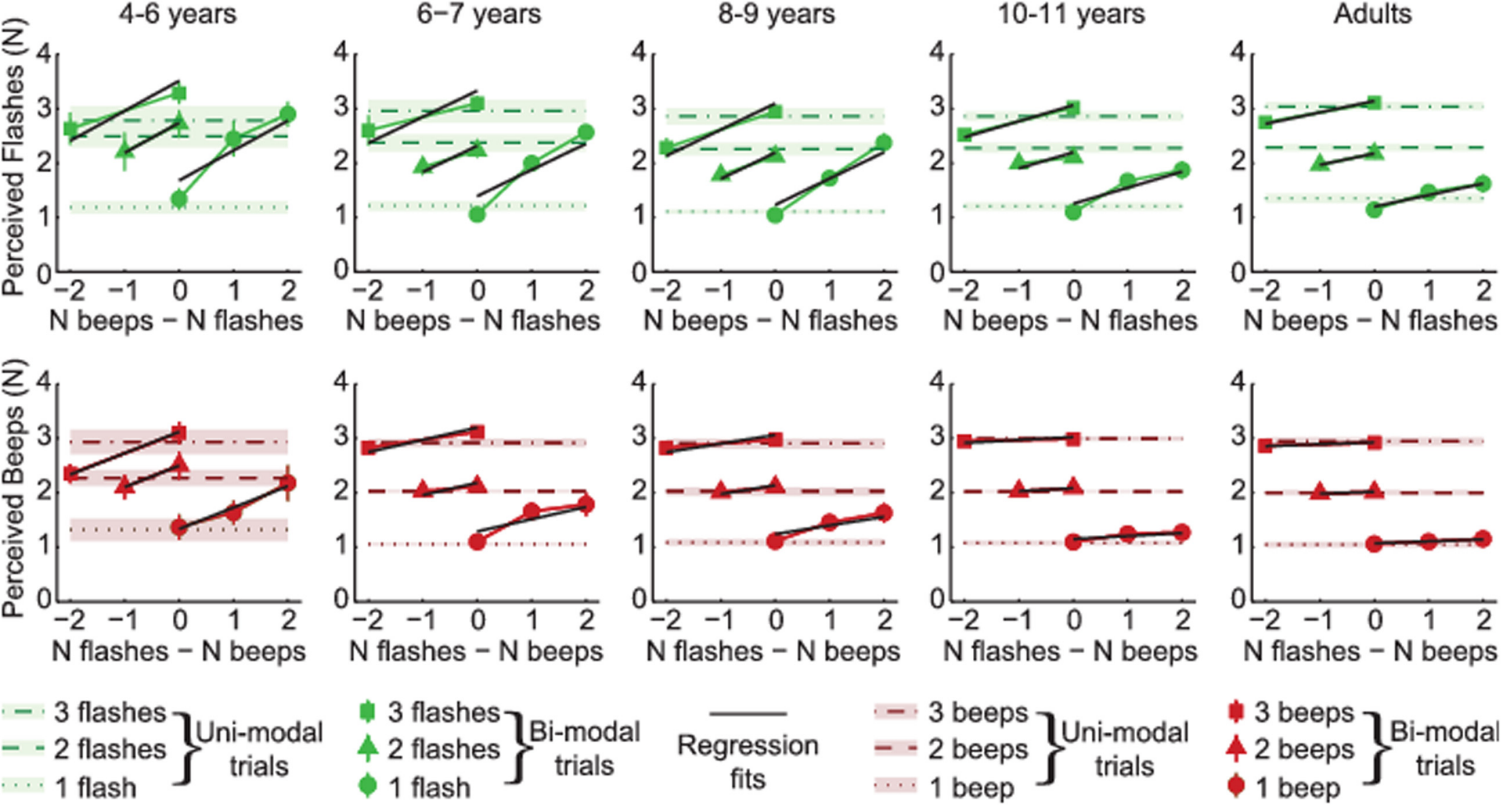
Summary of observers’ data. The reported number of flashes (upper row) and reported number of beeps (lower row). Each age group is shown by a separate column. Symbols give data from bi-modal trials. Horizontal dotted and dashed lines give responses on uni-modal trials, with the error bars / shaded regions giving ±1 SE across observers for bi- and uni-modal data, respectively. The influence of audition on vision (top row) or of vision on audition (lower row) is characterised by the slope of the best fitting regression lines (black lines). Regressions were performed for individual observers and subsequently averaged (for illustration only).

Data from 76 observers, split into 5 age groups, are summarised in Fig 1. Observers reported either the number of flashes (upper panels, green) or the number of beeps (lower panels, red). On some (uni-modal) trials, only flashes or only beeps were presented (Fig 1: horizontal dotted and dashed lines). These were intermingled with bi-modal trials in which both flashes and beeps were presented; the number of beeps and flashes could be the same or different.

On each trial, observers were explicitly asked to report either the number of flashes or the number of beeps, whilst ignoring the other modality. In the absence of any cross-modal interactions, data for the bi-modal conditions would fall along horizontal lines: increasing or decreasing the number of events in the task-irrelevant modality would have no effect on subjects’ responses. However, for all age groups, observers were unable to ignore the irrelevant, non-focal stimulus. The influence, or weight of the task irrelevant cue can be quantified by the slopes of the regression lines shown in Fig 1. These regression lines were fit to the bi-modal data separately for each observer and modality: one slope parameter quantifies the influence of audition on vision (upper plots) and another quantifies the influence of vision on audition (lower plots).

For all groups, the reported number of flashes was significantly modulated by the number of simultaneously presented beeps (one-sample *t*-tests against 0, all *p*<0.01). Likewise, the reported number of beeps was significantly affected by the number of flashes (all *p*<0.05). However, it is clear from Fig 1 that the size of this cross-modal interaction depended on which modality was being reported: task-irrelevant beeps had a much larger effect on the number of reported flashes than vice versa. The mean weight for audition, when reporting flashes, *w*_*VA*_ (as defined by the regression coefficients) was 0.38. The mean weight given to vision when reporting the number of beeps (*w*_*AV*_) was significantly smaller: 0.14 (main effect of modality: F_1, 71_ = 57.6, *p*<0.001).

We can ask whether the relative influence of the two modalities is predicted by their relative reliability, in line with standard models of optimal integration. In general, audition is more reliable than vision for temporal tasks such as the one employed here [15], and is therefore given more weight when integrated with vision, when observers are required to make temporal judgements. We can estimate the reliability of visual and auditory signals from the variance of observers’ responses on uni-modal trials. Across observers, audition was indeed more reliable than vision (mean variance for vision, $\sigma_{V}^{2}=0.36$, for audition, $\sigma_{A}^{2}=0.18$).

**Fig 2:**
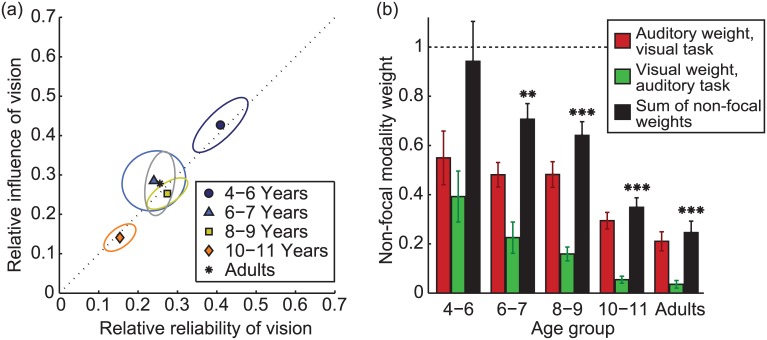
Sensory weights. (a) The relationship between the relative reliability of vision and the relative influence of vision, for each age group. It is clear that the relative reliability of vision predicts its relative influence for all groups. Covariance ellipses give 1SE around the mean. Note that the relative reliability of audition, *rr*_*A*_, and the relative weight for audition, *rw*_*A*_ can be calculated in an analogous way such that *rr*_*A*_ = 1 − *rr*_*V*_ and *rw*_*A*_ = 1 − *rw*_*V*_. Thus, the relative reliability of audition predicts the relative weight for audition in exactly the same way as for vision. (b) Sensory weights for non-focal modalities. Red bars give the weight given to (task-irrelevant) auditory information when reporting the number of flashes, while green bars give the visual weight when estimating the number of beeps. Black bars show the amount of integration, as quantified by the sum of the weights given to non-focal cues. Asterisks show the groups for which this sum is significantly less than 1 (one-sample *t*-tests).

Fig 2a summarises the relationship between the relative reliability of vision (*rr*_*V*_), as estimated from the uni-modal responses, and the relative weight of vision (*rw*_*V*_), as estimated from the bi-modal responses, where:

$$rr_{V}=\frac{1/\sigma_{V}^{2}}{\left(1/\sigma_{V}^{2}+1/\sigma_{A}^{2}\right)}\text{ and }rw_{V}=\frac{w_{AV}}{w_{AV}+w_{VA}},$$

where *w*_*AV*_ is the weight given to vision when reporting the auditory stimulus, and *w*_*VA*_ is the weight given to audition when reporting the visual stimulus. The relative influence of the two modalities is well predicted by their relative reliability for all groups (Fig 2a) and this relationship is significant across all individual observers (*r* = 0.54, *p*<0.001). It also reaches significance within the youngest (*r* = 0.77), middle (*r* = 0.67) and 4^th^ (*r* = 0.57) age groups (all *p*<0.05).

Interestingly, the strength of cross-modal interactions, as indexed by the sum of the non-focal cue weights, decreased substantially with age (main effect of age group: F_4, 71_ = 16.0, *p*<0.001; see the black bars in Fig 2b). If participants used the same weights for visual and auditory information, irrespective of the task (report beeps vs. report flashes), then the sum of the non-focal weights would be 1 (dashed line, Fig 2b). This is the prediction under standard models of full integration, e.g. [1, 2]. Conversely, if participants gave more weight to the focal, task relevant modality whilst down-weighting the task irrelevant one, the average weight would be less than 1, and would be 0 if observers were able ignore the task-irrelevant modality completely.

For the youngest observers only, the weights given to visual (red) and auditory (green) information did not vary according to whether observers were reporting beeps or flashes; the sum of the non-focal weights did not differ from 1 (see asterisks in Fig 2b). In other words, 4–5 year olds did not show any selectivity in reporting the focal, rather than non-focal modality. All other groups, however, showed partial cross-modal interactions: the reported number of flashes was dominated by visual information, whilst the reported number of beeps was dominated by auditory information, i.e. more weight was given to the focal modality, and the task-irrelevant modality was increasingly ignored as a function of age. Note that, in order to avoid floor or ceiling effects, the inter-stimulus interval (ISI) decreased with age (see Methods). A decrease in ISI (with all other factors constant) would be expected to increase uncertainty about the number of events, and thus increase the interaction between the sensory signals. For example, in the limiting case, the double flash illusion [16] will be eliminated with a large enough ISI. Note that the opposite pattern is seen here as a function of age—the influence of the non-focal cue *decreases* with age, despite the reduction in ISI. In other words, if the ISI had been more similar across age groups we would expect this age-related decrease in cross-modal interactions to be even larger.

Ernst [17] and Ernst & Di Luca [6] have described a variant of the standard Bayesian optimal integration model that allows partial integration, similarly to the behaviour described above. The model incorporates a ‘coupling prior’ that determines the strength of integration. This coupling prior represents the observer’s prior knowledge about the joint distribution of the two signals, i.e. the extent to which flashes and beeps tend to be correlated in the world and thus the probability that the visual and auditory signals contain redundant information. Under partial integration, variance in sensory estimates is no longer minimised, and in this sense, the integration strategy is no longer optimal. However, partial integration considers both precision (inverse variance) and accuracy (mean error). Under partial integration with potentially biased sensory signals, expected bias (inaccuracy) in the final estimates is reduced relative to full integration, whereas expected variance will be greater than under full integration. Partial integration can thus be described as optimal in the sense that it represents a balanced compromise between precision and accuracy [6]. Note that the standard, full integration model represents a special case of the partial integration model, in which signals are assumed to be accurate and the coupling prior is infinitely narrow (see Model 1: Partial Integration).

**Fig 3:**
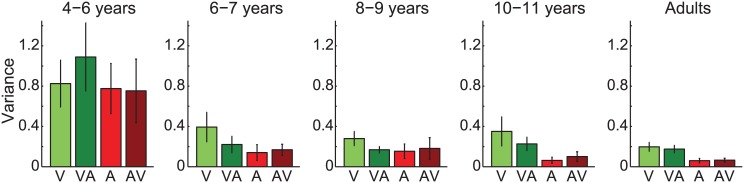
Response variance as a function of age. Lighter bars give response variance, averaged across uni-modal conditions for vision (V; green) and audition (A; red). Variance for bi-modal conditions is shown by darker bars for visual responses (VA; green) and auditory responses (AV; red). Error bars give ±1SE across observers.

The partial integration model has previously provided a good account of adults’ cross-modal integration in a similar, discretized task [5]. Similarly to standard models of integration, the partial integration model predicts a reliability benefit (i.e. a reduction in variability) when information is combined across modalities. However, the magnitude of this benefit is proportional to the strength of integration, and the reliability of the bi-modal estimates will not necessarily exceed the reliability of *both* of the component uni-modal estimates. However, bi-modal reliability should always improve relative to estimates from the focal modality alone. In other words, it predicts that our observers will be more reliable in reporting the number of flashes when both visual and auditory information is available, than from vision alone. We should expect a similar reliability improvement for bi-modal, relative to uni-modal auditory estimates. Furthermore, if all our observers were integrating optimally (i.e. following the Bayesian partial integration model) then the youngest group would show the largest bi-modal improvement in reliability, given that they show the strongest cross-modal interactions (black bars, Fig 2b). Fig 3 compares response variance for uni- and bi-modal estimates for the 5 age groups.

The youngest group shows no evidence of improved reliability in bi-modal, relative to uni-modal conditions for either vision or audition; their responses do not appear to reflect optimal sensory integration. All other groups show reduced variance in bi-modal, relative to uni-modal conditions for visual responses: compare dark and light green bars in Fig 3, this approached significance for 6–7 year olds and 8–9 year olds (*p* = 0.08, *p* = 0.07 for groups 6–7 and 8–9 years, all other age groups *p*>0.1, from paired *t*-tests). However, there is little difference between uni- and bi-modal variance for auditory responses (red bars). Many older observers had little response variance in the uni-modal and bi-modal auditory conditions, and, given the discretized nature of the task, we must be somewhat cautious in using our observers’ response variance as an accurate estimator of their underlying sensory noise. Furthermore, older observers gave very little weight to vision in bi-modal auditory conditions (as indicated by the small slopes in the lower plots of Fig 1) and thus the predicted improvement under optimal partial integration is very small. For these reasons, we evaluated whether the Bayesian partial integration model provides a good account of observers’ behaviour by calculating the likelihood of each observer’s data given this model. We compared it with two other candidate models in which observers do not integrate auditory and visual information, but instead (sub-optimally) switch between them, responding on each trial according to only visual or only auditory information.

### Modelling

**Fig 4:**
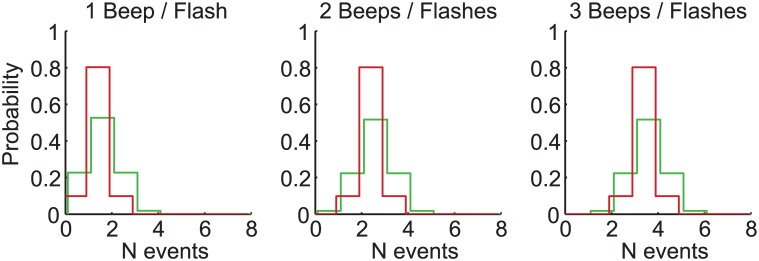
Uni-modal likelihoods. The best-fitting uni-modal likelihoods for vision (green) and audition (red), averaged (for illustration only) across all observers; they have been slightly horizontally offset for visibility. The spread of the likelihood (i.e. the inverse reliability) is fixed as a function of the number of events, but differs between vision and audition. On average, vision was less reliable than audition (*σ*_*V*_ = 0.772, SE = 0.052; *σ*_*A*_ = 0.488, SE = 0.058).

**Fig 5:**
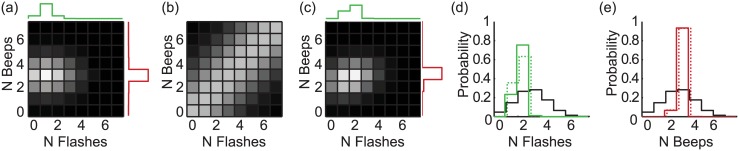
The partial integration model. (a) An example bi-modal likelihood, centred on 1 flash and 3 beeps. The uni-modal marginals are shown alongside. (b) The coupling prior, and (c) the bi-modal likelihood after combination with the coupling prior; the peak of the distribution has shifted towards V = A. (d) The visual marginal (dashed green) is multiplied by the prior over the number of events (black) to give the posterior probability distribution of the number of visual events (solid green). (e) The posterior distribution for audition (solid red), given the prior over the number of events (black). Note that to allow easy comparison across the three models, the prior over the number of events is shown as a sequential step after the coupling prior is applied and the subsequent marginals are estimated. The two priors could equivalently be combined and applied in a single step. All plots show the averaged model fit across the set of observers (N = 36) who were best characterised by the PI model, as determined by comparing the likelihood of the data, given each of the three models.

**Fig 6:**
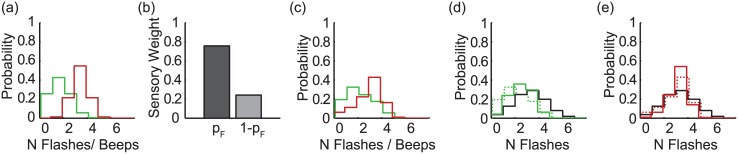
Focal switching model. Example (a) Uni-modal visual (green) and auditory (red) likelihoods. (b) On visual trials, the observer samples from the visual estimator with probability *p*_*F*_, and from the auditory estimator with probability 1 − *p*_*F*_. On auditory trials, these probabilities, or weights are reversed. The resultant likelihoods are shown in (c). Similarly to the PI model, posterior distributions (d, e, solid lines) are created by combining these likelihoods with a prior (black) over the number of events. All plots show the averaged model fit, averaged across the set of observers (N = 25) who were best characterised by the Focal Switching model.

**Fig 7:**
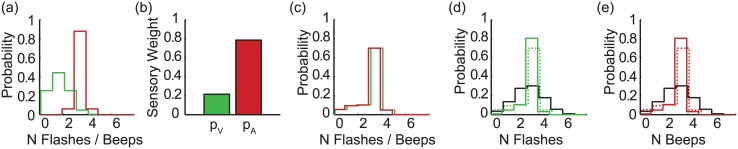
Modality switching model. (a) Unimodal visual (green) and auditory (red) likelihoods. (b) On both visual and auditory trials, the observer samples the visual estimator with probability *p*_*V*_, and the auditory estimator with probability 1 − *p*_*V*_. The resultant likelihoods (slightly offset for visibility) are shown in (c). Posterior distributions (d, e, solid lines) are created by combining these likelihoods with a prior (black) over the number of events. All plots show the averaged model fit across the set of observers (N = 15) who were best characterised by the Modality Switching model.

Three models were compared: (i) Partial Integration, (ii) Focal Switching, and (iii) Modality Switching. Note that these were evaluated separately for each observer; averaged fits are shown in Figs 4–7 for illustration only. For all three models, because the number of events can take integer values only, noise distributions, and the resultant uni-sensory likelihoods were approximated by discretised Gaussians, i.e. the probability of a sensory estimate equal to *x*, is given by $p(x)=a\text{exp}(-\frac{{\left(x-\mu \right)}^{2}}{2\sigma^{2}})$, {*x* ∈ℤ | ≥ 0} where *a* is a normalising constant. Noise distributions were centred on the true stimulus value, *μ*, but differed in variance, *σ*^2^, for vision and audition (see Fig 4).

In addition, alternative models were evaluated including the Causal Inference model [18, 19], models with logarithmic coding of number (corresponding to skewed likelihoods in linear space), and those that allowed likelihoods to be biased and / or to vary in reliability as a function of the number of events (beeps or flashes). These other models provided an inferior account of the data, as described in the supporting information file: S1 Text.

The three models (Partial Integration, Focal Switching, Modality Switching) differ in the way that sensory information from vision and audition interact:

#### Model 1: Partial integration

The partial integration (PI) model [6, 17] is a variant of the widely used, standard Bayesian model in which sensory estimates are always fully integrated, e.g. [1, 2, 20]. A coupling prior determines the strength of integration: a flat prior results in no integration—visual flashes and auditory beeps are processed independently. Conversely, a 2D prior given by the unity line V = A is equivalent to the standard full integration model, which would result in observers always perceiving an equal number of flashes and beeps. Fig 5 depicts an intermediate case—the best-fitting coupling prior, averaged across all observers whose responses followed the PI model.

The coupling prior is given by $p(x_{V},x_{A})=a\text{exp}(-\frac{{\left(x_{V}-x_{A}\right)}^{2}}{2\sigma_{C}^{2}})$, {(*x*_*V*_,*x*_*A*_)∈ ℤ(*x*_*V*_,*x*_*A*_)≥0} where *x*_*V*_ and *x*_*A*_ are visual and auditory sensory estimates, $\sigma_{C}^{2}$ is the variance of the coupling prior and *a* is a normalising constant. All three models include a prior over the number of events—models without this prior were inferior (see supporting information: S1 Text). The prior distribution over the number of events, *s*, is given by $p(s)=a\text{exp}(-\frac{{\left(s-\mu_{P}\right)}^{2}}{2\sigma_{P}^{2}})$, {*s*∈ℤ| *s* ≥ 0} where *μ*_*P*_ and $\sigma_{P}^{2}$ are the mean and variance of the prior and *a* is a normalising constant. For the PI model, one consequence of this prior is that responses from uni-modal trials are more biased than responses from congruent bi-modal trials; the prior has more influence on uni-modal trials when the available sensory information is less reliable.

Following the standard Bayes’ formulation, the posterior probability of a particular pair of visual and auditory estimates, ${\hat{s}}_{V=i,}{\hat{s}}_{A=j}$, is given by multiplying likelihoods and priors:

$$p({\hat{s}}_{V}=i,{\hat{s}}_{A}=j)\propto \text{exp}(-\frac{{\left(i-\mu_{V}\right)}^{2}}{2\sigma_{V}^{2}})\text{exp}(-\frac{{\left(j-\mu_{A}\right)}^{2}}{2\sigma_{A}^{2}})\text{exp}(-\frac{{\left(i-j\right)}^{2}}{2\sigma_{C}^{2}})\text{exp}(-\frac{{\left(i-\mu_{P}\right)}^{2}}{2\sigma_{P}^{2}})\text{exp}(-\frac{{\left(j-\mu_{P}\right)}^{2}}{2\sigma_{P}^{2}})$$

where *μ*_*V*_ and *μ*_*A*_ give the true number of flashes and beeps, respectively. On any single trial, the observer reports only one estimate: either the number of flashes, or the number of beeps. The posterior probability of a particular response is given by summing over all non-focal response estimates, i.e. finding the marginal probability distributions.

The PI model has 5 free parameters: (i) visual reliability, (ii) auditory reliability, (iii) width of coupling prior, *σ*_*C*_ (iv) mean *μ*_*P*_ and (v) spread *σ*_*P*_ of the prior over the number of events (beeps or flashes). Fitted values of these parameters are summarised in Table 1.

**Table 1:** Fitted parameters for the three models.

Parameter		Partial Integration (N = 36)		Focal Switching (N = 25)		Modality Switching (N = 15)	
		*μ*	*σ*	*μ*	*σ*	*μ*	*σ*
1	Visual variability, *σ*_*V*_	0.5433	0.1749	0.9943	0.5329	0.949	0.5322
2	Auditory variability, *σ*_*A*_	0.3441	0.2473	0.729	0.7767	0.4328	0.1832
3	Coupling prior spread, *σ*_*C*_	5.31E+05^a^	1.97E+06	—	—	—	—
	Focal weight, *p*_*F*_	—	—	0.7579	0.2151	—	—
	Visual weight, *p*_*V*_	—	—	—	—	0.2146	0.1534
4	Prior over n events, mean, *μ*_*P*_	2.6168	1.3401	2.7884	1.023	2.6467	0.8162
5	Prior over n events, std, *σ*_*P*_	1.3705	1.1015	1.3723	0.6105	1.2695	0.8499
-Log likelihood		70.15	45.17	143.67	71.42	97.41	35.80

The values of the 5 parameters for each model that maximise the likelihood of observers’ data. For each of the three models, the mean and standard deviation of the parameter values are shown, across all observers whose data were best fit by that model.

^a^The distribution of fitted values for this parameter was very skewed across observers; the median value was 0.79.

#### Model 2: Focal switching

Rather than integrating visual and auditory information, observers might stochastically switch between the two—sometimes responding according to the visual information, and sometimes according to audition [7, 8]. In the focal switching model, the distribution of responses depends on whether the observer is reporting the perceived number of flashes or beeps: observers select their estimate from the focal modality with probability *p*_*F*_ and the non-focal cue with probability (1-*p*_*F*_). Equivalently, this strategy produces a combined, bi-modal likelihood that is a weighted sum of the two uni-modal likelihoods: if the observer is reporting flashes, the likelihood of a particular estimate, *i*, is given by a weighted average of the probabilities of that estimate given the visual and auditory likelihoods: $p(\hat{s}=i)=p_{F}p({\hat{s}}_{V}=i)+(1-p_{F})p({\hat{s}}_{A}=i)$, where $\hat{s}$ is the estimate from the combined, bi-modal likelihood, and ${\hat{s}}_{A}$ and ${\hat{s}}_{V}$ are estimates from the visual (focal) and auditory (non-focal) likelihoods.

The model has 5 free parameters: (i) visual reliability, (ii) auditory reliability, (iii) focal probability and (iv) the mean and (v) variance of the prior over the number of events (beeps or flashes). Note that on conflict trials (in which n flashes ≠ n beeps), such as the example shown in Fig 6, cross-modal interactions produce an *increase* in variance, relative to the uni-modal likelihoods.

#### Model 3: Modality switching

In the Modality Switching model, observers again stochastically sample from auditory and visual information. However, in this model observers sample visual information with probability *p*_*V*_, and auditory information with probability *p*_*A*_ (where *p*_*V*_ + *p*_*A*_ = 1), irrespective of the focal modality. Under Modality Switching, $p(\hat{s}=i)=p_{V}p({\hat{s}}_{V}=i)+p_{A}p({\hat{s}}_{A}=i)$, where $\hat{s}$ is an estimate from the combined, bimodal likelihood, and ${\hat{s}}_{V}$ and ${\hat{s}}_{A}$ are estimates from the visual and auditory likelihoods. In other words, for bi-modal conditions with a given number of flashes and beeps (e.g. 1 flash and 3 beeps, as shown in Fig 7), the model predicts the same pattern of responses, irrespective of whether the observer is reporting beeps or flashes. However, similarly to the Focal Switching model, when the visual and auditory estimates differ, bi-modal response variance will be increased, relative to variance in uni-modal conditions.

For older observers, responses were strongly modulated by the response modality, with more weight given to the focal cue (see Fig 2b). However, this was not the case for the youngest observers, who gave similar weight to vision and audition, irrespective of which was focal (compare the leftmost pair of bars in Fig 2b). The Modality Switching model could, therefore, provide a good fit to younger observers’ behaviour.

### Modelling results

**Fig 8:**
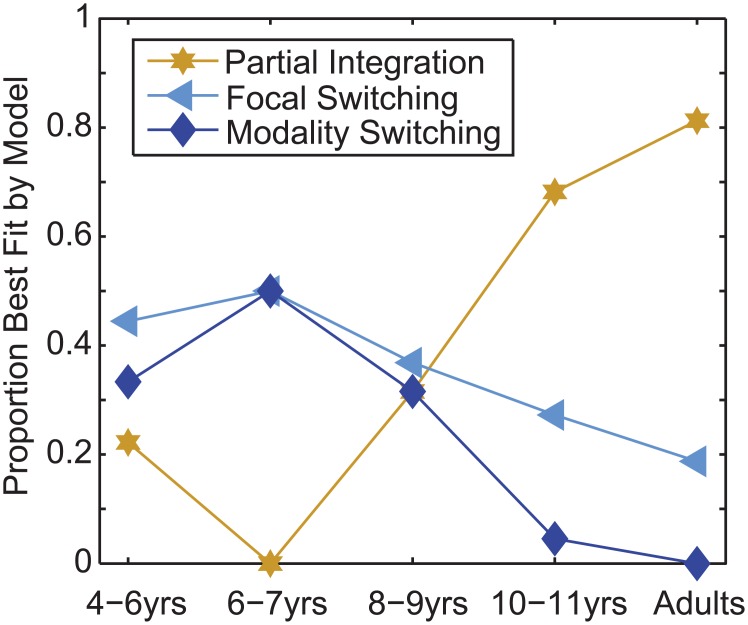
The best-fitting model of audio-visual interactions, as a function of age.

For each observer and each model, the values of the 5 free parameters were found (Matlab: fminsearch) that maximised the joint likelihood of the observer’s data across all uni-modal and bi-modal conditions. To avoid the problem of local minima, 288 iterations of the search were performed, making use of the University of Southampton’s IRIDIS High Performance Computing facility, with initial values uniformly sampled from the multidimensional space of plausible parameters. Fig 8 shows how multisensory interactions change as a function of age. Observers in the two youngest groups were best described by the switching models. Children aged 8–9 years were evenly split, and by 10 years the majority of observers followed the partial integration model.

As the three different models have common parameters, (visual and auditory noise, and the mean and variance of the prior over the number of events) we can consider how the best fitting values of these change as a function of age. Recent work [21] suggests that children as young as 7 quickly learn the statistics of a stimulus set and bias their estimates towards the mean. In the current study, knowledge of the stimulus statistics would be represented within the prior over the number of events. As participants learn these statistics we might expect both the mean and standard deviation of the prior to decrease, as participants learn that only a small numbers of beeps and / or flashes are presented. The youngest group had the weakest prior (largest standard deviation) of all age groups; this parameter varied significantly as a function of age (F_4, 71_ = 3.03, *p*<0.05). Post hoc comparisons showed that the youngest group had a significantly weaker prior than the 6–7 and 8–9 year olds (*p*<0.05 from independent *t*-tests, after correction for multiple comparisons), no other comparisons were significant. Whilst the fitted prior for youngest group also had the largest mean, this did not vary significantly across groups. This provides some evidence that the youngest group may have been slower to learn the stimulus statistics.

As might be expected from the raw response variance shown in Fig 3, the fitted visual and auditory noise parameters also varied as a function of age (F_4, 71_ = 6.8, *p*<0.001; F_4,71_ = 6.9, *p*<0.0001, for *σ*_*V*_ and *σ*_*A*_, respectively). Visual noise decreased monotonically with age, auditory noise decreased across each age group pair that shared a common stimulus ISI. Posthoc *t*-tests showed that, based on the fitted noise parameters, the youngest group was significantly more variable in both vision and audition than all other groups *p*<0.01, after corrections for multiple comparisons). In the current paradigm ISI decreased with age (in order to broadly equate task difficulty). With a fixed ISI we would expect a larger increase in visual and auditory temporal acuity as a function of age.

## Discussion

A simple task was used to investigate the developmental trajectory of audio-visual integration. Importantly, we evaluated three different models of integration that together provide a good account of sensory integration behaviour at all stages of development. Key findings emerged:

1. Observers of all ages combined visual and auditory information using appropriate sensory weights, as determined by the relative reliability of visual and auditory signals (see Fig 2a). This contrasts with previous findings in which younger observers relied entirely on one sensory estimate, and not always the most reliable one [10]. In that study, children viewed the front of the object, while touching the reverse side. It is has been suggested that the spatial offset and / or the fact that the active hand was obscured from view, prohibited cross-modal interactions in younger participants [22]; adults show reduced integration when sensory information is spatially offset [23]. Studies in which the sensory signals are aligned have found evidence for cross-modal interactions, i.e. switching behaviour, but not optimal integration, in younger children [7, 22].
2. Integration was automatic, and younger children were far less able to ignore task-irrelevant sensory information than older observers. This is in broad agreement with recent work which suggests that 7–10 year olds are unable to ignore irrelevant visual stimuli when performing an auditory spatial discrimination task [24]. In the current study, the youngest observers fully combined auditory and visual information, such that for bi-modal stimuli they perceived the number of flashes and the number of beeps to be the same. Older observers’ behaviour was well modelled by a partial integration model in which the coupling of visual and auditory information was relatively weak.
3. Optimal integration, as indexed by increased reliability, emerged by 10 years—this is broadly in line with previous findings [7, 10, 12]. Before this age, model comparison suggests that observers do not integrate sensory information, but stochastically sample from each modality. Our finding suggests that optimal integration of auditory and visual signals develops at a similar to age to integration across and within other modalities. Why did a previous study fail to find optimal audio-visual integration of temporal signals [13]? The study used a temporal bi-section task in which observers estimated which of two empty intervals was longer. Subsequent work has shown that for this type of task, with empty intervals, observers integrate auditory and visual information to optimally estimate the time points at the ends of the interval, rather than integrating duration per se [4]. With filled intervals, it is likely that optimal integration of duration estimates would be found with children aged 10 or so, as it is in adults [4].

Sensory integration has the potential to provide benefits for virtually all of our everyday activities—precision is improved by combining redundant information sources either within or across modalities. An obvious question remains unanswered—why does this ability fail to appear until around 10 years? One proposed explanation is that the lack of integration is beneficial during early childhood, and facilitates recalibration [10, 12]. During this period of growth and sensory development, constant sensory recalibration is required in order to maintain accurate (unbiased) perceptual estimates. Recalibration requires the estimation of inter-sensory conflict—if this were only possible in the absence of integration, i.e. by keeping sensory estimates separate, then the developing sensory system might forego integration in favour of recalibration. The importance of cross-sensory interaction for sensory calibration and development is supported by studies in populations with sensory impairments—congenital visual deficits appear to have a detrimental effect on the precision of haptic estimates and vice versa [25, 26].

Studies with adult observers, however, suggest that integration and recalibration are not mutually exclusive. For example, when glasses distort the relationship between binocular disparity and depth, the perceptual system recalibrates accordingly, whilst continuing to integrate binocular disparity with other depth cues [27]. Moreover, the sensory system adapts relatively quickly (within hours) when sensory statistics change [17, 28–30]. In fact, recalibration and integration both rely on establishing the correspondence between signals—identifying which signals are redundant and only integrating (or recalibrating) when they arise from the same source. It might be that younger children find this correspondence hard to learn [31]. In the current study, observers were told to ignore one modality—adults were able to do this to a large extent, whereas children were sub-optimal in the sense that cross-modal influences were larger, even though vision and audition were discrepant on the majority of trials. A previous study also found that the effect of auditory beeps on the reported number of flashes was larger in children than adults [32]. However, that study did not use a design that allowed optimal integration to be evaluated.

One recent study using a visual-proprioceptive reaching task did find some evidence of optimal integration, as evidenced by a reliability benefit, in children as young as 4–6 years [33]. However, this was only for the subset of observers who showed similar reliability for visual and proprioceptive estimates. Sub-optimal behaviour in other observers was attributed to inappropriate weighting. However, because the study did not include cue-conflict conditions, precise estimation of cue weightings was not possible. Our data suggest that, at least for the current task, the lack of integration shown by our observers was not due to a failure to weight the available signals appropriately.

In summary, the current work suggests that optimal integration does not emerge until around 10 years. Model comparison suggests that before that age, observers switch between the information provided by the two modalities, but do so in accordance with their relative reliabilities. This behaviour does result in responses centred on optimal values, but variance is larger than under optimal integration. In contrast with previous work, our younger observers did not rely on a single modality—in fact they were less able to ignore task-irrelevant information. Instead, they instead showed stronger, mandatory cross-sensory interactions than older observers.

## Methods

### Stimuli

**Fig 9:**
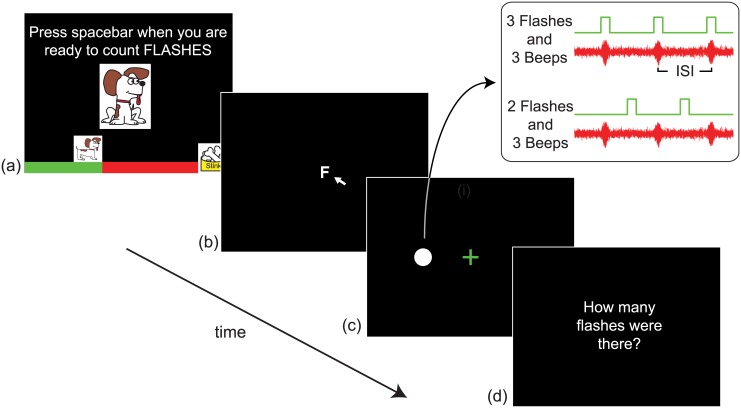
Trial schematic. (a) Instructions were shown at the start of each block of trials, and the voice of Stinker the dog gave the same instructions. A progress bar showed Stinker getting closer to his treats, as more trials were completed. (b) Either an ‘F’ or ‘B’ in the centre of the screen reminded the participant of the task. (c) After the letter was clicked, flashes, beeps or both were presented. The inset shows an example congruent trial (upper) and conflict trial (lower). (d) The participant was prompted to respond. An image of Stinker the dog appeared every few trials, with Stinker’s voice offering words of encouragement or comments, e.g. ‘You’re great’, or ‘I’m hungry’.

Visual stimuli were white discs subtending 2.2 degrees of visual angle (dva) at the viewing distance of 45 cm with a luminance of 196 cd/m^2^. These were presented briefly (1 flash = 16.7msec), centred at 5.7dva to the left or right (randomly across trials) of a central fixation cross on an otherwise black screen. Auditory stimuli were presented via small speakers placed either side of the screen. These consisted of short beeps: 440Hz tones in a Gaussian temporal envelope of σ = 21msec. To reduce the reliability of the auditory stimuli, these beeps were embedded in continuous white noise [4]. As in previous studies, sequences of flashes and beeps were temporally aligned [14], see Fig 9a.

The spacing between events (the ISI) was varied as a function of age group, as determined by pilot work. This was done to roughly equate task difficulty across groups such that floor or ceiling effects were avoided: pilot work showed that a fixed ISI across groups resulted in floor effects for the youngest group (such that the number of perceived events did not systematically increase as a function of the true number of events) and / or ceiling effects in the adult group (no response errors). For children in school years 1–3 (infant school; age 4–7 years) beeps and / or flashes were spaced by an ISI of 200msec. For junior school children (school years 4–7; age 7–11 years) the ISI was 167msec and for adults it was 117msec.

### Procedure

All participants were given detailed instructions, and completed 8 practice trials in which they reported flashes (4 trials) or beeps (4 trials). When counting flashes, subjects were told to ignore any beeps and vice versa. To help with motivation and concentration, participants were told that they needed to help Stinker the dog count beeps or flashes in order to get his treats. At the start of each block of experimental trials, Stinker appeared on the screen and instructed the participant to ‘count the flashes’ or ‘count the beeps’.

Each trial began with an ‘F’ or a ‘B’ presented at the screen’s centre to remind participants of the current task. To ensure fixation, participants were required to use the mouse to click this letter. The letter then changed to a green fixation cross and the sequence of flashes and / or beeps was presented. Each sequence consisted of 0–3 beeps and 0–3 flashes, such that the trial could be uni-modal (only beeps or only flashes), bi-modal congruent (equal number– 1, 2, or 3 –of flashes and beeps) or bi-modal conflict (the number of beeps and flashes differed by 1 or 2). Uni-modal and bi-modal trials were randomly intermingled, but trials were blocked by focal modality (i.e. report flashes, or report beeps).

Participants gave their response on each trial by selecting the appropriate number on the keyboard (1–9); they were not told the maximum or minimum number of possible beeps or flashes. To keep the task duration within the concentration span of the child participants (approximately 20 minutes, based on pilot work), infant school children completed 140 trials (2 modalities: judging beeps or flashes) x 10 conditions (3 uni-modal, 7 bi-modal) x 7 repetitions. Junior school children completed 8 repetitions (160 trials) and adults completed 12 repetitions (240 trials).

### Participants

We report data from 76 observers (60 children, 16 adults). A further 5 children from the 4–6 age group were excluded who failed to complete the task and / or could not reliably count up to 3. To check for counting ability / task comprehension, we used leave-one-out cross validation to compare regression models for each observer’s data, to ensure that the reported number of events across uni-modal and bi-modal congruent trials increased significantly as a function of the true number of events.

Children were *a priori* divided into four age groups, by splitting the infant and junior school children at the midpoint of each age range, such that all children within a group were given the same stimulus set (i.e. the same ISI). The resultant 5 groups were (i) ‘4–6 Years’: Range: 4 years 9 months to 6 years 3 months, *n* = 9, 6 males (ii) ‘6–7 Years’: Range 6yrs 5m to 7yrs 8m, *n* = 11, 7 males (iii) ‘8–9 Years’: 7yrs 9m to 9 yrs 8m, *n* = 19, 9 males (iv) ‘10–11 Years’: 9yrs 11m to 11yrs 5m, *n* = 22, 8 males and (v) ‘Adults’: Range 18–41 years, *n* = 16, 8 males). The study was approved by the ethics committee at the University of Southampton and all participants gave informed consent. Parents / guardians gave consent on behalf of their children and children also provided consent on the day of the experiment.

## Supporting Information

S1 Text Description and evaluation of alternative models. The supporting information provides a description and evaluation of alternative models of observers’ data. First, we apply the Causal Inference model [18], with three different decision rules [19]. Second, we test whether participants’ responses are more consistent with log-based coding of number. Finally, we show that more complex models, such as those in which the likelihoods can be biased, or noise changes as a function of the number of events, do not provide a significantly better fit to the data.
(PDF)
