# Supplementary material for: The Development of Audio-Visual Integration for Temporal Judgements
Source: PLoS Comput Biol. 2016 Apr 14;12(4):e1004865. doi: 10.1371/journal.pcbi.1004865 (PMC4831745; doi:10.1371/journal.pcbi.1004865)
Supplement: S1 Text — The supporting information provides a description and evaluation of alternative models of observers’ data. First, we apply the Causal Inference model [18], with three different decision rules [19]. Second, we test whether participants’ responses are more consistent with log-based coding of number. Finally, we show that more complex models, such as those in which the likelihoods can be biased, or noise changes as a function of the number of events, do not provide a significantly better fit to the data. (PDF) [file pcbi.1004865.s001.pdf]

S1 Text

The Development Of Audio-Visual Integration For Temporal Judgements

Wendy J. Adams

Supporting Information: Description and evaluation of alternative models

Here we consider alternative models that might account for observers' data. First, we apply the Causal Inference model [1], with three different decision rules [2]. Second, we test whether participants' responses are more consistent with log-based coding of number. Finally, we show that more complex models, such as those in which the likelihoods can be biased, or noise changes as a function of the number of events, do not provide a significantly better fit to the data.

Causal Inference model

In addition to the Partial Integration model, the Causal Inference model has also been proposed to describe behaviour in situations where two sensory signals are not fully integrated to produce a single, multisensory estimate. The model is described in detail, with full equations, elsewhere [1, 2]. Therefore it is only described in brief here, as applied to the current experimental paradigm. In order to evaluate the Causal Inference model, the noise distributions, posterior, and response distributions are modelled by simulating a large number of experimental trials for each stimulus condition. Accordingly, uni-modal noise distributions were generated for each condition of the current experiment by simulating 10,000 trials. For each simulated trial, noisy visual and auditory sensory estimates (x_V and x_A) were generated by sampling from normal distributions centred on the true number of flashes and beeps, with standard deviations σ_V and σ_A, respectively. Because the number of events can only take integer values of zero or more, the noise-corrupted sensory estimates were rounded to the nearest integer. Simulated trials resulting in negative estimates were discarded. The resultant noise distributions are shown in Fig S1a.

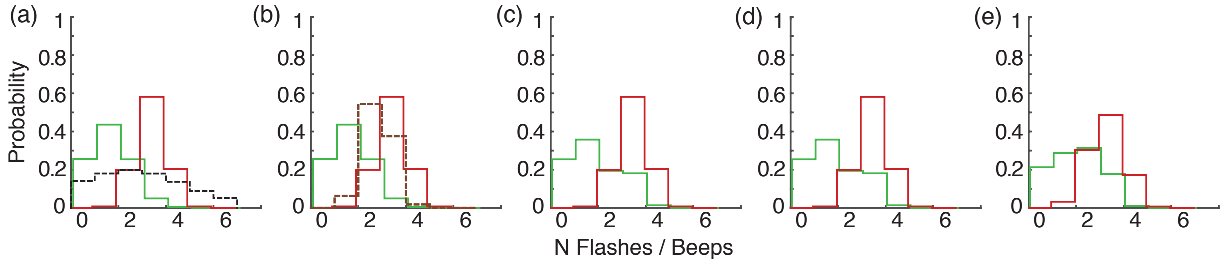

Fig. S1. The Causal Inference model. (a) Visual (green) and auditory (red) noise distributions for an example condition with 1 flash and 3 beeps, generated via 10,000 simulated trials using, for illustration, the average fitted parameters across all observers. The prior over the number of

events is also shown (dashed black). (b) Visual (green) and auditory (red) estimates given separate causes. The probability distribution of common cause estimates is shown by the dashed brown line. (c, d, e) Response distributions for the estimated number of flashes (green) or beeps (red), given each of the 3 decision rules: (c) Model averaging, (d) Model selection, and (e) Probability Matching.

In parallel to the Partial Integration and Switching models, the Causal Inference model also includes a prior probability distribution over the number of events, defined by μ_P and σ_P, (see dashed black line in Fig S1a). However, rather than employing a coupling prior to determine the degree of sensory integration, the Causal Inference model considers two discrete causal structures. Under the hypothesis that the visual and auditory estimates had a common cause (C=1), the sensory signals are fully integrated such that the estimated number of flashes ($\hat{s}_{V,C=1}$) is equal to the estimated number of beeps ($\hat{s}_{A,C=1}$). This common cause estimate is given by a weighted average of x_V, x_A and μ_P, where the weights are given by $1/\sigma_V^2$, $1/\sigma_A^2$ and $1/\sigma_P^2$, respectively. The probability distribution of common cause estimates, produced by 10,000 simulated trials is shown by the dashed brown line, Fig S1b.

Under the second hypothesis, the sensory estimates (x_V and x_A) arose from separate, independent causes. In this case, the estimated number of flashes ($\hat{s}_{V,C=2}$) can differ from the estimated number of beeps ($\hat{s}_{A,C=2}$), with these estimates given by a weighted average of x_V and μ_P, or x_A and μ_P, respectively. Distributions of these separate cause estimates are also shown in Fig S1b.

The response produced on each trial depends upon the posterior probability of a common cause (vs. separate causes), and the decision rule. The posterior probability of a single source is calculated, via Bayes' rule, from the conditional probability of the sensory estimates, given a common cause, and the prior probability of a common cause, i.e. p_{common}, or p(C=1): $p(C=1 \mid x_V, x_A) \propto p(x_V, x_A \mid C=1)p(C=1)$. The posterior probability of separate causes is calculated in an analogous fashion, where the prior probability of separate causes, p(C=2) is equal to 1 – p_{common}.

Three different decision rules (described below) have been proposed to determine how a response is generated on each trial. The corresponding response distributions, from simulated trials, are shown in Fig S1(c-e).

(i) Model averaging. This is the optimal decision rule, if the goal is to minimise the expected mean squared error. The response is determined by a weighted average of the estimated number of flashes (or beeps) given common or separate causes, with weights given by the posterior probability of these two causal states, i.e.

$\hat{s}_V = \hat{s}_{V,C=1}p(C=1 \mid x_V, x_A) + \hat{s}_{V,C=2}p(C=2 \mid x_V, x_A)$, with a similar computation determining the auditory response, $\hat{s}_A$.

(ii) Model selection. On each trial, the response reflects either the estimate given a common cause, or the estimate given separate causes. The selection is determined by the posterior probabilities of the two causal states, if a common cause is more likely, given the sensory estimates, then the single cause estimate is selected, i.e.

$$\hat{s}_V = \hat{s}_{V,C=1} \text{ if } p(C=1 \mid x_V, x_A) > 0.5$$
$$\hat{s}_V = \hat{s}_{V,C=2} \text{ if } p(C=1 \mid x_V, x_A) \leq 0.5$$

(iii) Probability matching. Similarly to model selection, on each trial the response reflects either the common cause estimate or the separate causes estimate. However, under probability matching, this selection process is stochastic (as in the Switching models described above). The probability of selecting the common cause estimate is given by the posterior probability of a common cause; the probability of selecting the independent causes estimate is given by the posterior probability of separate causes. This can be implemented by sampling a value ζ, from a uniform distribution in the range 0 to 1, then

$$\hat{s}_V = \hat{s}_{V,C=1} \text{ if } \zeta < p(C=1 \mid x_V, x_A)$$
$$\hat{s}_V = \hat{s}_{V,C=2} \text{ if } \zeta \geq p(C=1 \mid x_V, x_A)$$

The Causal Inference model, as applied to the current experimental paradigm, has 5 free parameters: (i) visual variability, (ii) auditory variability, (iii) the prior probability of a common cause, (iv) the mean of the prior over the number of events, and (v) the standard deviation of this prior. For each of the three decision rule variants, and separately for each participant, the parameters were found (via Matlab: fminsearch) that maximised the joint likelihood of the participant's data across all uni-modal and bi-modal conditions. To avoid the problem of local minima, 288 search iterations were performed, with initial values sampled from the multidimensional space of plausible parameters. The best fitting parameters are given in Table S1, which also shows the number of participants whose data were best described by each of the three decision rule variants of the model.

Of the three Causal Inference variants, the majority of participants were best fit by the Probability Matching decision rule. This is in broad agreement with previous work in adults [2]. The decision rule did not vary substantially or systematically as a function of age: within the three variants of the Causal Inference model, Probability Matching provided the best fit to the majority of participants in each age group.

Table S1. Maximum likelihood parameter estimates for the Causal Inference model.

Decision Rule variant of the Causal Inference model:		Model Averaging (N=21)		Model Selection (N=11)		Probability Matching (N=44)	
Parameter		μ	σ	μ	σ	μ	σ
1	Visual variability, σ_V	1.0582	0.6624	0.8681	0.7644	0.8616	0.7782
2	Auditory variability, σ_A	0.7255	0.6786	0.7281	0.8382	0.5430	0.5938
3	Common cause probability, p_{common}	0.6331	0.2403	0.3835	0.4052	0.6353	0.3858
4	Prior over n events, mean, μ_P	2.0505	1.8813	1.5952	1.0731	1.9622	2.0813
5	Prior over n events, std, σ_P	2.1386	0.4852	2.6952	0.5940	2.5121	0.8513
-Log likelihood		121.64	61.24	117.09	79.22	97.59	54.10

Causal Inference compared to Partial Integration and Switching Models

Because the Partial Integration, Switching, and Causal Inference models are matched in complexity (each with 5 free parameters) we can compare how well they account for participants' data by directly comparing the likelihood of the data given each model. Fig S2a shows the proportion of participants that is best fit by each of the six models considered thus far, as a function of age group. If we take the log likelihood of the data for each participant given the best fitting of the original three models (Partial Integration, Focal Switching, Modal Switching), and compare this with the best fitting variant of the Causal Inference models, we can see that the Causal Inference models provide a significantly worse fit to the data (t_{75}=-6.8, p=2x10^{-9}). The Causal Inference models provide a worse fit to the data, relative to the original 3 models for 63 of the 76 observers. This is illustrated in Fig S2b, which shows for each observer, the log likelihood of the best fitting of the original 3 models, against the log likelihood of the best fitting of the Causal Inference models.

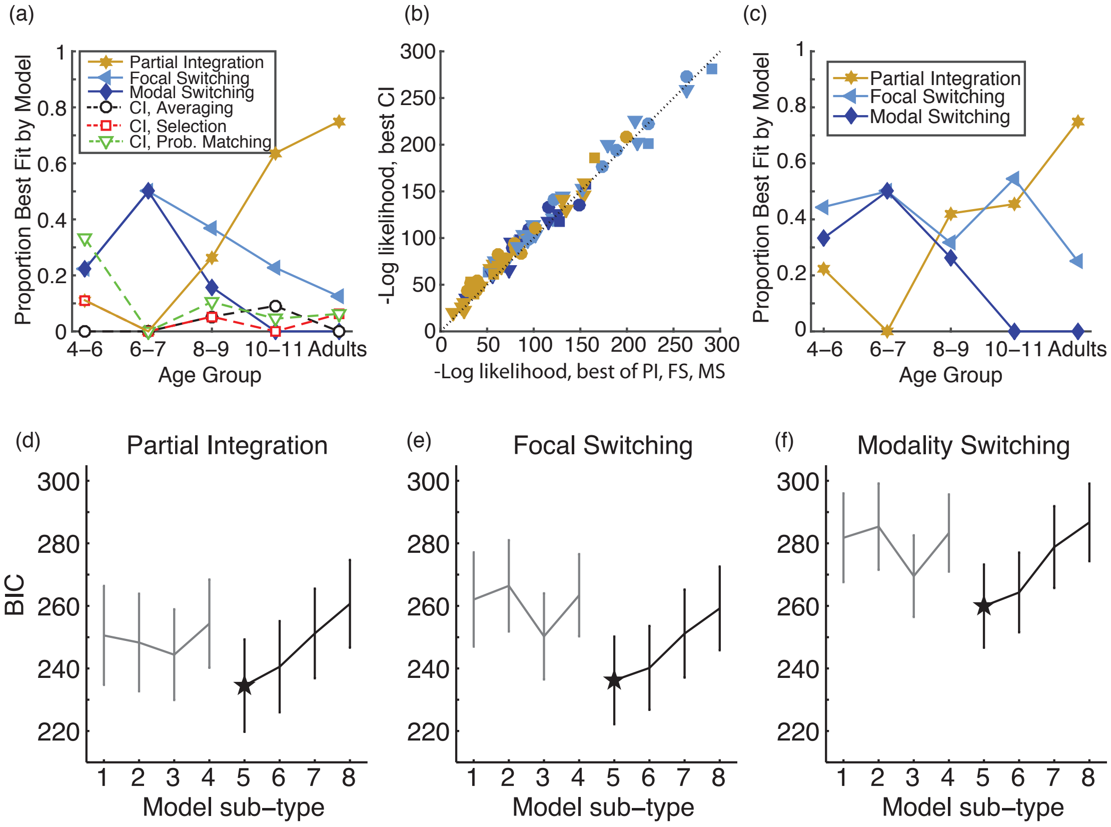

Fig. S2. Model Comparisons. (a) The proportion of participants best fit by each of the six models, as a function of age group. (b) For each observer, the log likelihood of the data given the best fitting of the Partial Integration, Focal Switching and Modality Switching models (x-axis) is shown – colour indicates the best fitting model. This is plotted against the log likelihood of the best fitting variant of the Causal Inference model – shape indicates the best fitting decision rule variant. (c) Results of logarithmic coding: The proportion of participants best fit by each of the three models, as a function of age, when uncertainty is Gaussian in log space. (d) For the Partial Integration model, (e) the Focal Switching model and (f) the Modality Switching model, the BIC is shown for each of the 8 sub-types, averaged across all 76 observers. The first four model sub-types (grey) are the same as the last four (black), other than the inclusion of a prior over the number of events for the latter. Error bars give ±1SE across observers. For all three types of model, sub-type 5 is optimal (lowest BIC), as indicated by the star symbols. Within each of the three model types, the BIC varied significantly across subtypes (one-factor repeated measures ANOVAs, $F_{7,525}=23.6$, $p<10^{-27}$; $F_{7,525}=32.2$, $p<10^{-36}$; $F_{7,525}=30.9$, $p<10^{-35}$ for the Partial Integration, Focal Switching and Modality Switching models, respectively) and sub-type 5 performed significantly better than all others (all $p<10^{-5}$, from paired *t*-tests, after Bonferroni corrections for multiple (7) comparisons), with the exception of sub-type 6, which was not significantly worse than 5 within either the Focal or Modality Switching models.

Logarithmic coding of number

Previous work, e.g. [3], has suggested that number may be coded logarithmically, such that the perceptual difference between successive integers becomes increasingly small. For the current paradigm, this would suggest that sensory likelihoods should be

positively skewed. To determine whether our participants appear to be estimating number in this way, the Partial Integration, Switching and Causal Inference models were re-evaluated with skewed likelihoods and prior distributions, such that p(log(frequency)) was Gaussian distributed. Overall, this change made little difference to the general pattern of results, with younger participants best modelled by the Switching models and older observers following the Partial Integration model (Fig S2c). In comparison to the linear versions of the models, Logarithmic coding produced a slightly, but insignificantly worse fit to observers' data, in terms of the average log likelihood (t_{75}=-1.49, p>0.05). It is possible that logarithmic coding of number becomes more apparent when the number of events increases beyond the subitizing range.

Models of varying complexity

We also compare models with and without a prior over the number of events. For each type of model: Partial Integration (PI), Focal Switching (FS) and Modality Switching (MS), we evaluated 8 different model sub-types of varying complexity. For each sub-type, the parameter values that maximised the likelihood of each observer's data were found (Matlab: fminsearch). Multiple iterations of the search procedure were completed for each observer and subtype, with different initial parameter values, sampled uniformly from the multi-dimensional space of plausible values.

Model sub-types 1 – 4 did not include a prior over the number of events.

(i) Sub-type 1 (3 free parameters): As in the optimal models, noise distributions are unbiased, with reliability characterised by a separate variance parameter for vision (σ_V) and audition (σ_A). The third parameter gives the width of the coupling prior (σ_C, model PI), or the sampling probability (p_F or p_V, models FS, MS).

(ii) Sub-type 2 (7 free parameters): Likelihoods are unbiased, but reliability varies as a function of both the number of events and modality (vision, audition; σ_{Vi}, σ_{Ai}, i =1, 2 or 3). The seventh parameter gives the width of the coupling prior or the sampling probability (σ_C, p_F or p_V).

(iii) Sub-type 3 (9 free parameters): Noise distributions can be biased (μ_{Vi}, μ_{Ai}, i =1, 2 or 3 give the means). Reliability varies as a function of modality (σ_V, σ_A), but is fixed across 1, 2 or 3 events. The ninth parameter gives the width of the coupling prior or the sampling probability (σ_C, p_F or p_V).

(iv) Sub-type 4 (13 free parameters): Noise distributions can be biased, and reliability varies as a function of the number of events and the modality (μ_{Vi}, μ_{Ai}, σ_{Vi}, σ_{Ai}, i =1, 2 or 3). The final parameter describes the coupling prior or sampling probability (σ_C, p_F or p_V).

Sub-types (v - viii) were identical to (i - iv), but also included a prior over the number of events, characterised by 2 additional parameters (μ_P and σ_P). Sub-type (v) was the optimal model described in the main text. Because these models varied in complexity (but were not all nested), they were compared using Bayesian Information Criterion (BIC): see Fig S2 (d-f) and associated figure legend.

In addition, we tested analogous models in which the uni-modal likelihoods were characterised by binomial probability distributions, allowing negative skew. These provided a worse fit to the data.

References

1. Koerding KP, Beierholm U, Ma WJ, Quartz S, Tenenbaum JB, Shams L. Causal Inference in Multisensory Perception. Plos One. 2007;2(9). doi: 10.1371/journal.pone.0000943. PubMed PMID: WOS:000207455800022.

2. Wozny DR, Beierholm UR, Shams L. Probability Matching as a Computational Strategy Used in Perception. Plos Computational Biology. 2010;6(8). doi: 10.1371/journal.pcbi.1000871. PubMed PMID: WOS:000281389500004.

3. Merten K, Nieder A. Compressed Scaling of Abstract Numerosity Representations in Adult Humans and Monkeys. Journal of Cognitive Neuroscience. 2009;21(2):333-46. doi: 10.1162/jocn.2008.21032. PubMed PMID: WOS:000262909700010.
